# Kinematics of Photoisomerization Processes of PMMA-BDK-MR Polymer Composite Thin Films

**DOI:** 10.3390/polym12061275

**Published:** 2020-06-03

**Authors:** Qais M. Al-Bataineh, A. A. Ahmad, A. M. Alsaad, I. A. Qattan, Areen A. Bani-Salameh, Ahmad D. Telfah

**Affiliations:** 1 Department of Physical Sciences, Jordan University of Science & Technology, P.O. Box 3030, Irbid 22110, Jordan; qalbataineh@ymail.com (Q.M.A.-B.); sema_just@yahoo.com (A.A.A.); areensalameh92@gmail.com (A.A.B.-S.); 2 Department of Physics, Khalifa University of Science and Technology, P.O. Box 127788, Abu Dhabi 127788, UAE; issam.qattan@ku.ac.ae; 3 Leibniz Institut für Analytische Wissenschaften-ISAS-e.V., Bunsen-Kirchhoff-Straße 11, 44139 Dortmund, Germany; telfah.ahmad@isas.de; 4 Hamdi Mango Center for Scientific Research (HMCSR), the Jordan University, Amman 11942, Jordan

**Keywords:** polymerized nanocomposite thin films, optical data storage, UV-light sensors, photoisomerization processes, trans-isomer, cis-isomer

## Abstract

We investigate and report on the kinematics of photoisomerization processes of polymer composite thin films based on azo dye methyl red (MR) hosted in polymethylmethacrylate (PMMA) incorporated with Benzyl dimethyl ketal (BDK) as a photo-initiator. Understanding photoisomerization mechanisms is crucial for several optical applications such as Read/Write/Erase (WRE) optical data storage media, UV light Read/Write heads, and UV light sensors. The as-prepared polymer composite thin films are characterized using UV–Vis spectroscopy. Furthermore, Fourier transform infrared spectroscopy (FTIR) and scanning electron microscope (SEM) are employed to investigate the optical, chemical, and morphological properties of trans- and cis-states of PMMA-BDK-MR polymer composite thin films. The presence of the azo dye MR in the composite is essential for the efficient performance of the *cis ↔ trans* cycles through illumination ↔ thermal relaxation for Write/Read/Erase optical data storage and UV-light sensors. Moreover, UV–Vis and FTIR results confirm the hysteresis cycle of *trans-* and *cis-*states and that PMMA-BDK-MR thin films may be regarded as potential candidates for successful Write/Read/Erase optical data storage and UV-light sensors. In addition, the morphology of the thin film surface is investigated by SEM technique. The SEM images indicate that uncured surfaces of PMMA-BDK-MR thin films are inhomogeneous compared with the corresponding surfaces after curing. The transformation from inhomogeneous surfaces to homogeneous surfaces is attributed to the polymerization of thin films by UV curing.

## 1. Introduction

**Figure 1:**
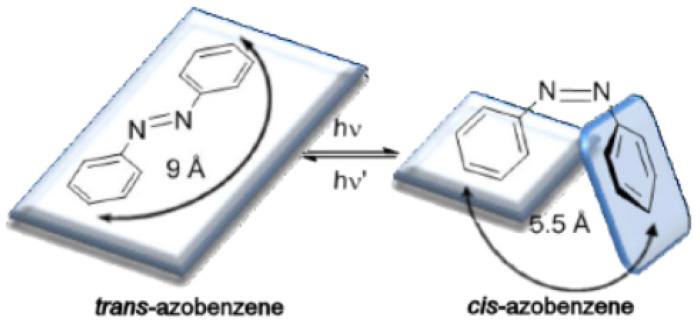
Photoisomerization process of azobenzene [10].

The performance of the lowermost electronically excited states of conjugated chromophore azobenzene has attracted the attention of chemists performing experimental and theoretical works to explicate and interpret the mechanism of double-bond photoisomerization. The composites containing chromophore groups [>A=B<] have gained evident awareness due to their potential applications in optical data storage devices, optical switching, photonic devices, polarization holography, and optical sensors [1,2,3,4,5,6,7,8,9,10,11]. The azo-benzene dyes are made up of organic molecules that take the shape of odorless rings interconnected by a conjugated chromophore azo group (−N=N−) together with heterocyclic systems. At present, azo-benzene dyes dominate 60% of the dyes market worldwide [12,13,14]. The photochromic properties of azo-benzenes have attracted great interest due to the isomerization of the (−N=N−) double bonds that occurs readily in the presence of a light source [15,16,17]. Like a C=C double bond, the azo-benzenes have two geometric isomers, *trans* and *cis*, around the N=N double bond. Energetically, the *trans* isomer of azobenzene dye is 10–12 kcal mol^−1^ more stable than the *cis* isomer [18]. The barrier energy of photo driven state in thermal equilibrium at room temperature is ~23 kcal.mol^−1^, indicating that the *trans* isomer is the principal isomer in low-illuminated region [19]. A well-known feature of all azo-benzenes is the proficient and revocable photoisomerization between a thermally stable *trans*-state and a metastable *cis*-state upon illumination with appropriate photon energy. Interestingly, a *cis*-*trans* isomer is attainable under thermal relaxation process [20,21]. It has been reported that *trans*-azobenzene converts to *cis* isomer by irradiation with UV light [20,21]. The photoisomerization reaction is reversible and the *trans* isomer is recuperated when the *cis* isomer is exposed to light of wavelength 400–450 nm, or to a proper thermal excitation [20,21,22]. The mechanisms of photoisomerization of the *trans-cis* isomer and thermally induced reverse *cis-trans* isomer in hybrid polymers plays a major role in elucidating physical, electric, structural, and optical properties of azo-benzenes [22,23,24]. The photo driven *trans*-*cis* isomerization process of polymers is forecast to be important in obtaining a deeper insight into the functioning of optical data storage devices, heat, and light sensors. The *trans-cis* and *cis-trans* cyclic behavior enable the azo-dye composites to be key potential candidates in Write/Read/Erase (WRE) cycles [20,21,25,26,27,28]. It has been reported that distance between the two carbon atoms in aromatic rings of azobenzene is shrunk from 9.0 Å in the *trans* form to 5.5 Å in the *cis* form as a result of photo-induced isomerization process as illustrated in Figure 1 [29]. The azo-benzenes that adopt *trans*-case are fundamentally flat and exhibits no dipole moment, while the azo-benzenes exhibiting *cis*-state assume an angular geometry and a significant dipole moment of 3.0 Debye [17].

A major part of this work is devoted to the study of the kinematics of photoisomerization processes of polymer composite thin films based on azo dye methyl red (MR) hosted in polymethylmethacrylate (PMMA) incorporated with Benzyl dimethyl ketal (BDK) as a photo-initiator. This composite is expected to play a crucial role in optical applications such as Read/Write/Erase (WRE) optical data storage media for the UV light Read/Write heads or UV light sensors. The optical, chemical, and morphological properties of the *trans*- and *cis*-states of PMMA-BDK-MR polymer composite thin films are typically characterized by UV–Vis spectroscopy, Fourier transform infrared spectroscopy (FTIR), and scanning electron microscope (SEM). To the best of our knowledge, we are not aware of any experimental study that has previously investigated the kinematics of photoisomerization mechanisms of PMMA-BDK-MR polymer composite thin films.

## 2. Theoretical Background

**Figure 2:**
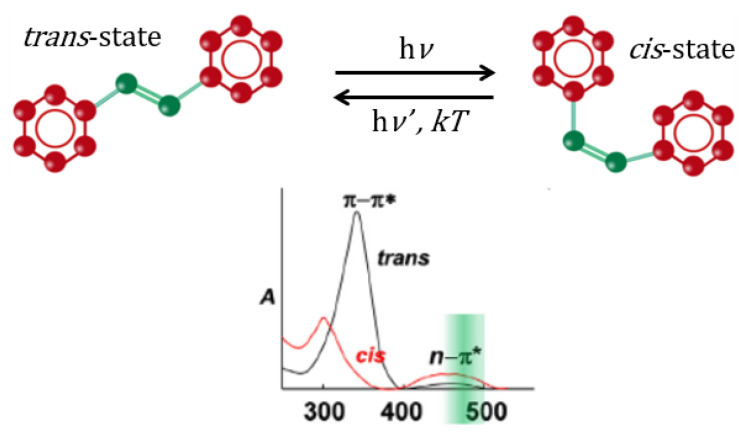
Reversible *trans-cis-trans* isomerization of azo-dyes derivatives and representative absorption spectra of *trans* and *cis* isomers [38].

The proficient and revocable photoisomerization between a thermally stable *trans*-state and a metastable *cis*-state under the illumination and thermal relaxation has been of prime importance for fundamental and practical perspectives [20,21]. *Trans*-phase of the isomers is thermally stable at room temperature [30,31] as shown in Figure 2. The major transitions that occur within the azo-benzene are mainly determined by the azo-benzenes substituents [32].

Usually, isomer undergoes a series of symmetric or antisymmetric non-breakable bending of one of the aromatic rings. Such twisting leads to prolongation and compression of the mono-molecule of the isomer, eventually causing successive variations in the absorption band of the azo-benzenes [33,34].

Figure 2 depicts the *trans-cis* cyclic conversions as well as the change in the bond length of the isomer mono-molecule. The bond length between the two carbon atoms in position four of the aromatic rings of azo-benzene shrinks during the isomerization process from 9.0 Å in the *trans* form to 5.5 Å in the *cis* form [29]. “Azobenzene undergoes *trans*–*cis* isomerization by $S_{1}\leftarrow S_{0}$ and $S_{2}\leftarrow S_{0}$ excitations and *cis*–*trans* isomerization by exciting into the $S_{1}$ or $S_{2}$ state” [35]. Isomerization in stilbene is attributed exclusively due to rotation with quantum yield equaling unity [36]. This new conformation is employed as a state of memory-WRITE or memory-READ state. Furthermore, *cis*-state can be transferred back to *trans*-state by means of thermal relaxation process. This feature is utilized for memory-ERASE state. The cycles of *trans* ↔ *cis* flip-flop states can be achieved by repeating the illumination ↔ thermal relaxation processes several times. Hence, the hysteresis process inspects the steadfastness of using the polymeric composites for memory-READ, WRITE, and EREASE states [37]. Azo-dyes tolerate photo driven reversible isomerization between *trans*-state and *cis*-state when exposed to photons with proper energy in their absorption bands [32].

## 3. Experimental Details

In this section the synthesis procedure of polymer composite thin films based on PMMA-BDK-MR is described in detail. Polymethylmethacrylate (PMMA) and Benzyl Dimethyl Ketal (BDK) were obtained from Sigma-Aldrich (St. Louis, MO, USA), while Methyl-Red (MR) of pH (4.2–4.6) powder was purchased from SCP. One g of PMMA, 1 g of BDK, and 1 g of MR were successively dissolved separately in 100 mL of pure tetrahydrofuran (THF), then the solutions were sonicated for 6 h to enhance the homogeneity. PMMA-BDK-MR polymer composite in the form of solution was obtained by mixing PMMA solution, BDK solution and MR solution in (3:3:1) volume ratio using a magnetic stirrer for 6 h. The mixed solution with the volume fractions was then filtered using a 0.45 μm Millipore filter before dip coating on the glass substrates. All films were prepared at standard conditions (i.e., room temperature of 25 °C and 1 atmospheric pressure). Polymer composite films were obtained by dipping the glass substrate in PMMA-BDK-MR polymer composite solution for 1 h to get a film of 300 nm thickness. The films were dried in air at 70 °C for at least 30 min to evaporate the solvent and organic residues.

The PMMA-BDK-MR thin films are initially exposed to UV-illumination by UV lamp of 366 nm with a power of 6 Watt for 0, 4, 8, and 12 s to study the transformation from *trans*-case to *cis*-case. The films are then treated with thermal relaxation process under atmospheric conditions at 90 °C for 20, 40, and 60 min to study the transformation from *cis*-case to *trans*-case. Consequently, it does not contribute to any permanent change in the optical constants, while BDK is activated by a transition within the molecular orbits of the >C=O group followed by an α-splitting to produce free radicals when it is irradiated by UV light [39].

## 4. Results and Discussion

### 4.1. Kinematics of Photoisomerization Processes

**Figure 3:**
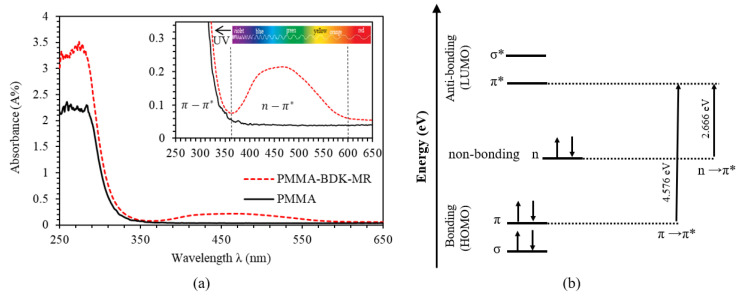
(**a**) Absorbance spectra of PMMA-BDK-MR (Polymethyl Methacrylate-Benzyl Dimethyl Ketal-Methyl Red) polymer composite thin film as a function of wavelength, and (**b**) HOMO and LUMO energy diagram of PMMA-BDK-MR polymer composite thin film concluded from the absorbance spectra shown in Figure 3a. The inset in Figure 3a shows an enlarged absorbance spectra in the spectral range 250–650 nm that illustrates the Absorbance peak associated with $n-\pi^{*}$ transition.

The main objective of this section is to study the kinematics of the transformation of PMMA-BDK-MR polymer composite thin films from the initial *trans*-state to *cis*-state with UV-illumination and thermally activated back transformation to *trans*-state. Figure 3 shows the absorbance spectra of PMMA-BDK-MR polymer composite thin film in initial *trans*-state compared with the absorbance spectra PMMA thin film as a function of wavelength. Absorption spectra of the azo isomers is usually characterized by double bond transitions, namely, the high-intensity band $\pi -\pi^{*}$ transition or $S_{1}$ excited state in UV range, and a weaker $n-\pi^{*}$ transition or $S_{2}$ excited state in visible range, compared to the PMMA which has a single bond transition, namely, the high-intensity band $\pi -\pi^{*}$ transition or $S_{1}$ excited state in UV range as shown in Figure 3a. The two transitions depend strongly on the azo-dyes substituents [32]. Obviously, the absorbance spectra of PMMA-BDK-MR thin film exhibits a $n-\pi^{*}$ peak in the spectrum region of (360–600) nm with absorption maxima in the visible range at 467 nm. This region represents the green, blue, and violet colors. This behavior is attributed to the existence of MR in the thin films that gives the films the reddish color. It is known that this red-colored thin film transmits red, yellow, and orange lights while it blocks other colors such as green, blue, and violet [40].

In molecular orbital theory, PMMA-BDK-MR molecules have two bonding molecular orbitals and two antibonding molecular orbitals, as seen in Figure 3b. The bonding molecular orbitals are lower in energy than the antibonding ones. So, π and σ are bonding molecular orbitals and $\pi^{*}$ and $\sigma^{*}$ are the antibonding molecular orbitals. Considering the molecular absorbing energy, it is important to describe the highest occupied molecular orbital (HOMO) and the lowest unoccupied molecular orbital (LUMO). When the electron absorbs enough energy, it jumps from the HOMO to the LUMO. Our results indicate that PMMA-BDK-MR molecules exhibit double bond transitions that can be labeled as high-intensity band $\pi -\pi^{*}$ transition in UV range at 271 nm. In this transition, electrons absorb 4.576 eV to jump from HOMO to LUMO. In the other transition described as a weaker $n-\pi^{*}$, transition occurs in the visible range at 465 nm, electrons absorb energy of 2.666 eV to move from nonbonding state to LUMO.

**Figure 4:**
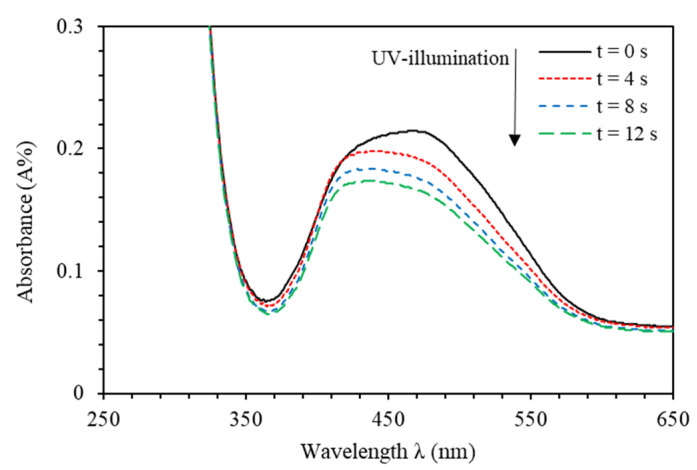
Absorbance spectra of PMMA-BDK-MR polymer composite thin film for various UV-illumination times, namely, natural *trans*-case, *trans/cis*-transformation case (1) after 4 s, *trans/cis*-transformation case (2) after 8 s and *cis*-state after 12 s.

The PMMA-BDK-MR thin films are initially exposed to UV-illumination for various time periods. The films are then treated with thermal relaxation process under atmospheric conditions for various times at 90 °C temperature. Figure 4 shows the absorbance spectra of PMMA-BDK-MR polymer composite thin films exposed to UV-illumination for different durations of time. As expected, thin films exhibit *trans*-state (black solid line, initial *trans*-state). The major absorption maxima of PMMA-BDK-MR thin film in the visible range is at 465 nm at initial *trans*-state with amplitude of 0.212. The thin film is then illuminated with energetic UV light of wavelength of 366 nm for 4, 8, and 12 s. As can be seen from Figure 4, the film acquires different absorbance band (350–600 nm) in the middle of the visible range with a blueshift in the head of the peak transferring the material from *trans*-state to *cis*-state, as expected. It can be seen that the transformation from *trans*-state to *cis*-state is not instantaneous transformation. Instead, the material transformation goes through several phases. The first stage of transformation occurs after 4 s of UV-illumination. This phase is characterized by the existence of major absorption maxima in the visible range at 460 nm with an amplitude of 0.191. The second stage of transformation occurs after 8 s of UV-illumination. This stage is featured by the existence of the major absorption maxima in the visible range at 459 nm with an amplitude value of 0.177. The final stage of transformation takes place after 12 s of UV-illumination. The final stage is characterized by the presence of the major absorption maxima in the visible range at 458 nm at final *cis*-state with an amplitude of 0.164. After 12 s of UV-illumination, neither the absorbance values change, nor do they exhibit blue shift.

Furthermore, Figure 4 indicates an absorption maximum of PMMA-BDK-MR thin film at ~465 nm (black-solid line) from the *trans*-isomer before it is exposed to UV illumination. Exposure to UV-light results in a noticeable reduction of the absorbance of the ~465 nm *trans*-isomer absorption band and the appearance of a second peak at ~458 nm (green-dashed curve) originated from the *cis*-isomer. Examination of the absorbance spectra of PMMA-BDK-MR thin film corresponding to the absorption band of a *trans-*isomer before and after illumination with UV-light indicates the existence of two isosbestic points appearing near 340 and 650 nm. Additionally, at room temperature, most of the MR molecules are expected to exist in a *trans* form in a PMMA-BDK matrix. Illumination of the molecules at this band causes the *trans-*form to isomerize to a *cis-*form. In other words, molecules illuminated with light of wavelength (650 nm≥λ≥340 nm) exhibit a decreasing absorbance as the illumination time increases. The absorption band in the wavelength range (650 nm≥λ≥360 nm) is attributable to the $S_{0}–S_{1}$ transition of the *cis-*form. This band also falls into the tail of a $S_{0}–S_{2}$ transition of the *trans-*form. The prolonged illumination does not change the shape of absorption spectrum, indicating that a photo-stationary state exists between *trans-* and *cis-* forms.

**Figure 5:**
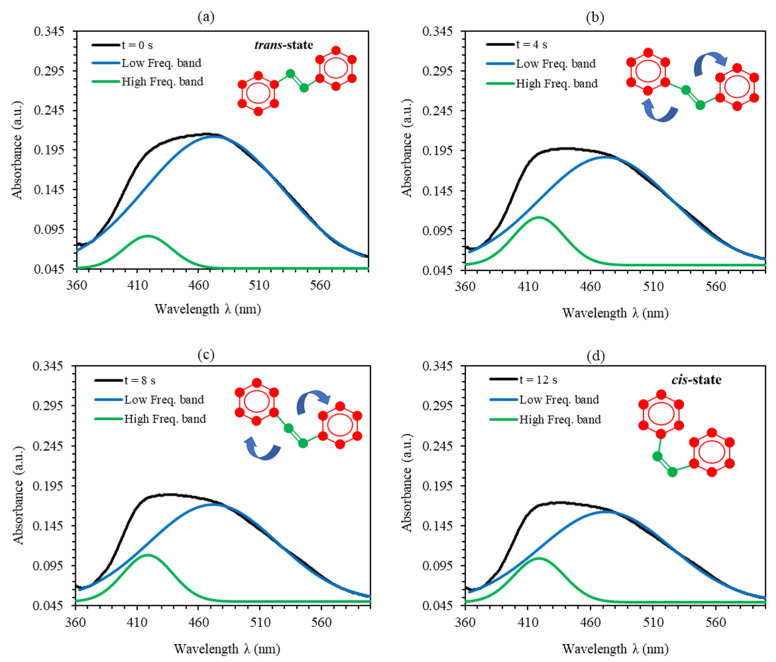
Double peaks fit for the absorbance spectra of PMMA-BDK-MR polymer composite thin film for various UV-illumination times, (**a**) Natural *trans*-case, (**b**) *trans/cis*-transformation case (1) after 4 s, (**c**) *trans/cis*-transformation case (2) after 8 s, and (**d**) *cis*-state after 12 s.

As can be clearly seen from Figure 4, each absorbance peak of PMMA-BDK-MR polymer thin film consists of more than one single absorption peak (frequency band). As the illumination time increases, the amplitude of the absorption peak of the band decreases and blue shifted confirming the transformation of thin films from *trans*-state to *cis*-state. Evidently, by increasing the time of UV-illumination, the development of configuration shapes of the observed peaks in Figure 4 endorses the occurrence of frequency bands consisting of two sub-peaks rather than one absorption peak. Additional piece of evidence for the existence of two bands comes from the fact that obvious shoulders are observed on the low-frequency side and high-frequency side (around 480 nm and 425 nm, respectively) of the absorbance curve. Figure 5a–d shows the major peaks in the visible spectrum of the absorbance fitted to two Gaussian peaks. The fitting converged to the deconvoluted line describing adequately the experimental data. Figure 5a depicts the major peak of PMMA-BDK-MR thin film in its initial *trans*-case in the visible spectrum of the absorbance fitted to two Gaussian peaks. The absorption spectra of PMMA-BDK-MR thin film exhibits two bands (high- and low-frequency) with maxima starting at around 418.38 and 472.68 nm and linewidths of 46.22 and 133.03 nm, respectively. The inset of Figure 5a displays the schematic diagram of the initial *trans*-case. The shape of the absorption spectrum changes as the UV-illumination time increases, as shown in Figure 5b–d, realized from *trans*-case to *cis*-case transformation.

**Figure 6:**
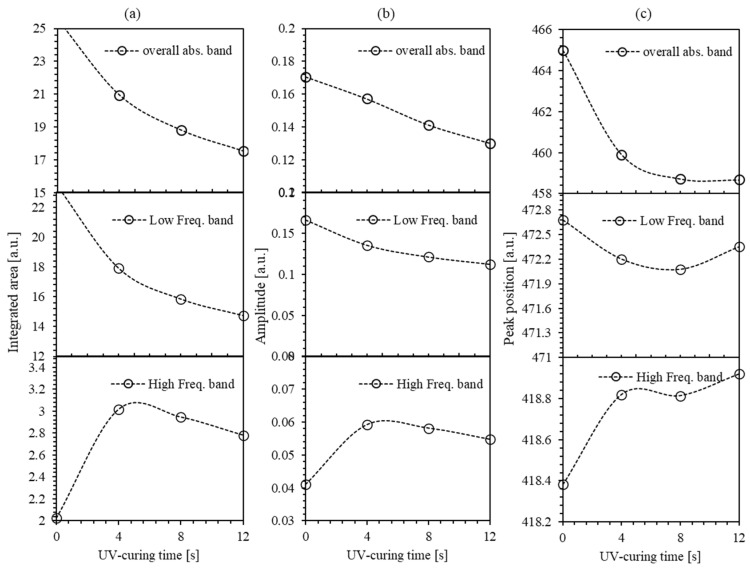
(**a**–**c**) Double peaks fit of the absorbance spectra of PMMA-BDK-MR polymer composite thin film for various UV-illumination times.

To obtain a deeper insight into the effect of time curing of the UV-illumination, we execute a more detailed quantitative analysis of the low and high-frequency spectral absorption bands of PMMA-BDK-MR polymer composite thin film along UV-illumination time curve. With a closer look at Figure 5, we can readily deduce the fine details of each peak, including the peak shifts, amplitude and the change in area under the spectral curve as demonstrated in Figure 6a–c. Interestingly, we explore the variations caused by UV-illumination process of the UV–Vis spectra during the transformation from *trans*-case to *cis*-case. The spectral analysis is performed in the spectral range containing the absorption peak at 465 nm. Since the two frequency bands are strongly overlapping, including their baselines, sensitive regression analysis approach with minimum correlation between the spectral parameters is needed in order to define the two distinct frequency sub-bands. In this regard, the main peaks in the visible range (as shown in Figure 5) are fitted to two parametrized Gaussian curves. It is well known that a single isolated frequency band related to the *n* → π* transition of azomethine-dye is best fitted by skewed functions [41,42]. In Figure 5, each main frequency band is represented by two sub-bands. The shape trend of each absorption peak (sub-band) varies with the time of UV-illumination obtained from the systematic deviation of the linewidth of the respected Gaussian function. The high-frequency sub-band acquired a red shift (Figure 6c). This shift designates a bathochromic change occurred due to the absorption of MR foreseeing that MR is a source of the H-bond donor or the source of the dissociated-intermediate H-bond donors [43,44]. This result proposed that unbonded hydroxyl was needed to perform the large bathochromic shifts shown in Figure 6c. Accordingly, the major absorption peak is predicted to occur due to the H-bond mutual interaction between the PMMA and the azo nitrogen via MR molecules. Both bands are shifted in the identical pattern despite additional difference in the absorbance-effected bonding demonstrating that H-bonds have substantial effect on different sites of MR molecules. During the UV-illumination process, the high-frequency band exhibits steady bathochromic shifts, indicating two explicit symmetries at the frameworks of the H-bonds associated with MR transformation. The accumulative absorbance maximum and low-frequency sub-band absorbance maximum do not shift confirming that the time-dependent transformation absorbance is performed via multiple steps rather than a single one [45]. This is because the transformation from *trans*-case to *cis*-case is a complex response to a blend of generic and specific solute–solvent interactions [41,46,47].

**Figure 7:**
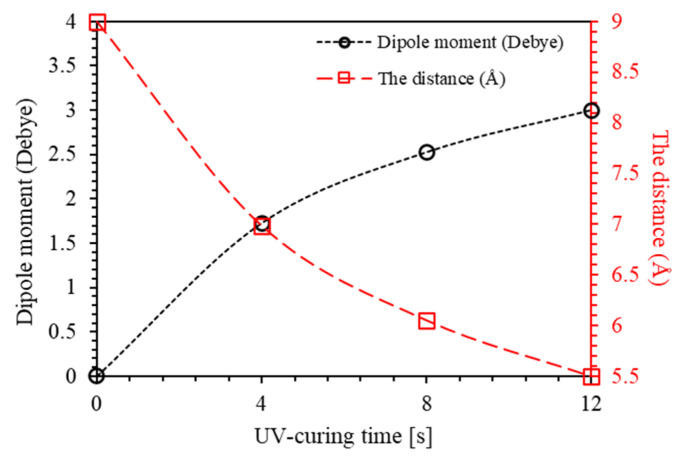
The distance between the two carbon atoms in position 4 of the aromatic rings and the dipole moment of MR in for PMMA-BDK-MR polymer composite thin film for various UV-illumination times.

A similar argument applies to the progression of the amplitudes of the absorbance peaks. The area under the absorbance peak represents the whole process rather than the peak amplitude. Figure 6a,b shows the amplitudes curve areas and the cumulative absorbance maxima obtained separately from the low-frequency band and the high-frequency band. It is known that the *trans*-case of the polymer thin film is almost flat with the distance between the two carbon atoms in position 4 of the aromatic rings of 9.0 Å and has no dipole moment, whereas, *cis*-case presents an angular geometry with the distance between the two carbon atoms in position 4 of the aromatic rings of 5.5 Å and a dipole moment of 3.0 Debye [17,29]. From these values and the area under the spectral curve (Figure 6a), the distance between the two carbon atoms in position 4 of the aromatic rings and the dipole moment of each stage are calculated and illustrated in Figure 7.

**Figure 8:**
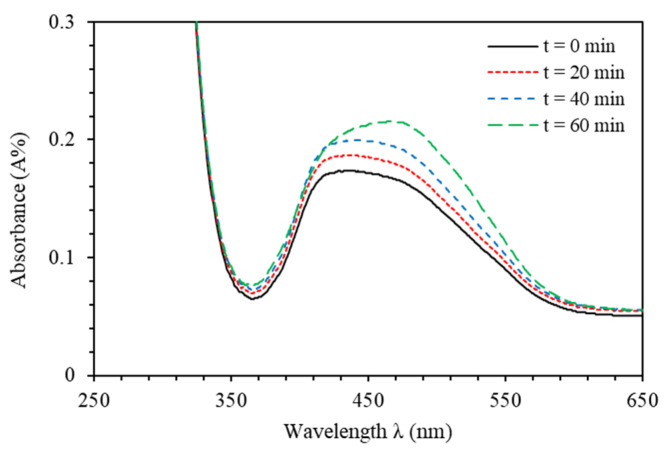
Absorbance spectra of PMMA-BDK-MR polymer composite thin film for various thermal process times: *cis*-case, *cis/trans*-transformation case (1) after 20 min, *cis/trans*-transformation case (2) after 40 min, and *trans*-state after 1 h.

Figure 8 shows the absorbance spectra of PMMA-BDK-MR polymer composite thin film for various thermal process times. After 12 s of UV-illumination, thin films are found to exhibit *cis*-state (black solid line). The major absorption maxima of PMMA-BDK-MR thin film in the visible range is at 458 nm with an amplitude of 0.177. The thin film is then treated with thermal relaxation process under atmospheric conditions at 90 °C for 20, 40, and 60 min. As can be seen from Figure 8, the film acquires different absorbance bands (350–600 nm) at the middle of the visible range with a redshift in the head of the peak transferring thin film from *cis*-state to *trans*-state, as expected. It can also be seen that the transformation from *cis*-state to *trans*-state is not instantaneous transformation. Rather, it transforms through several stages. The first stage of transformation is observed after 20 min of thermal process. The major absorption maxima in the visible range is at 459 nm with an amplitude of 0.184. The second stage of transformation is noticed after 40 min of thermal process. The major absorption maxima in the visible range is at 460 nm with an amplitude of 0.197. The final stage of transformation is obtained after 60 min of thermal process. The major absorption maxima in the visible range is at 465 nm at final *trans*-state with an amplitude of 0.215.

### 4.2. WRE Optical Data Storage Cycle

**Figure 9:**
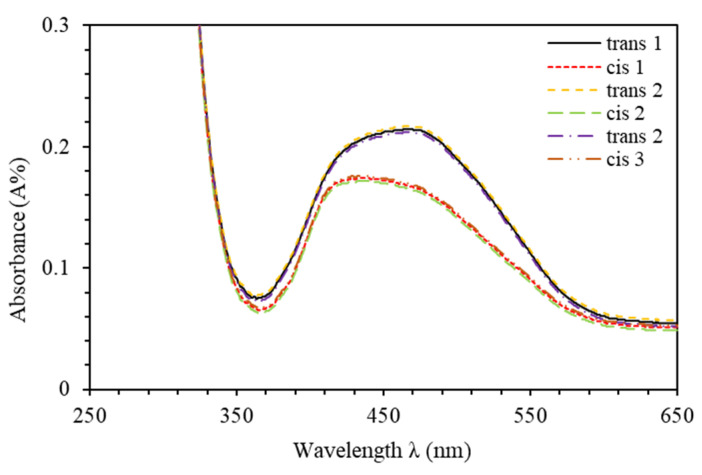
Absorbance spectra of PMMA-BDK-MR polymer composite thin film: Natural *trans*-case, *cis*-state after 12 s of UV-366 nm illumination and *trans*-state after annealing at 90 °C for 1 h.

**Figure 10:**
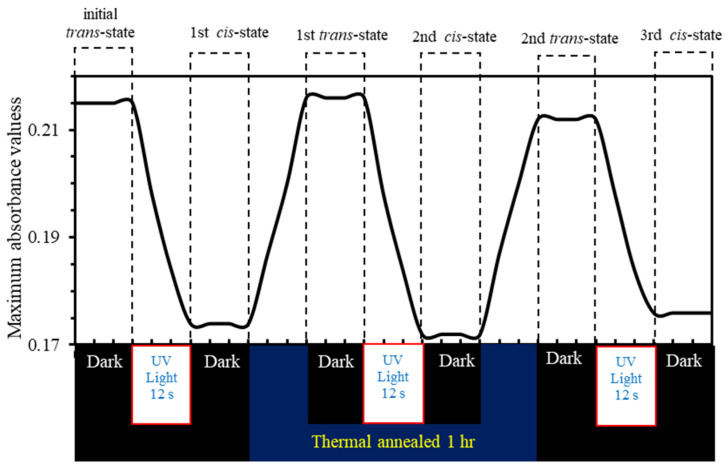
Maximum absorbance values through photochemical and thermal processes that transforms from *trans* to *cis* and vice versa of PMMA-BDK-MR polymer composite thin films.

**Figure 11:**
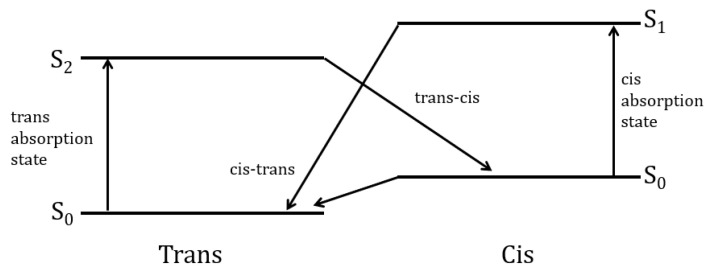
Four level diagram describing *trans*- and *cis*- isomerization.

Figure 1 and Figure 2 illustrate the photo-switching mechanism of MR occurred by a *trans-cis* isomerization process. Exposure of the *trans*-isomer to UV-light results in *trans*- to *cis*-isomerization. The subsequent *cis*-isomer would thermally/optically relax back to the *trans*-isomer. PMMA-BDK-MR thin films are exposed to optimized conditions of illumination. The films then undergo thermal relaxation process under atmospheric conditions for one hour at 90 °C temperature. Figure 9 shows the absorbance spectra of PMMA-BDK-MR polymer composite thin films. The figure illustrates the stages of photoisomerization. Firstly, it shows the absorption spectra prior to light illumination and heat treatment (black solid line, initial *trans*-state). The thin films are then illuminated by energetic UV-light of wavelength 366 nm for 12 s. As a result, the films exhibit a new absorbance band at the middle of the visible range shifting blue shift. This ratifies the transformation of the isomer from *trans*-state to *cis*-state (red dot-line, *cis*-state). The formation of the absorbance band in the visible range is attributed to the presence of MR in the PMMA-BDK-MR films. Lastly, the films undergo thermal annealed for one hour at 90 °C. This process transforms thin films back to *trans*-state (yellow dashed line, *trans*-state again). The *cis* to *trans* or *trans* to *cis* acts as “on” and “off” buttons in electrical circuits or “logic 1” and “logic 0” in the binary data storage media. The *cis* ↔ *trans* is periodically repeated as the photoisomerization/thermal relaxation process is repeated, confirming a reliable hysteresis loop, as illustrated in Figure 10. Figure 11 illustrates diagrammatically the four level model of *trans-* and *cis-* isomerization [21,28].

### 4.3. Fourier Transform Infrared Spectroscopy (FTIR)

**Figure 12:**
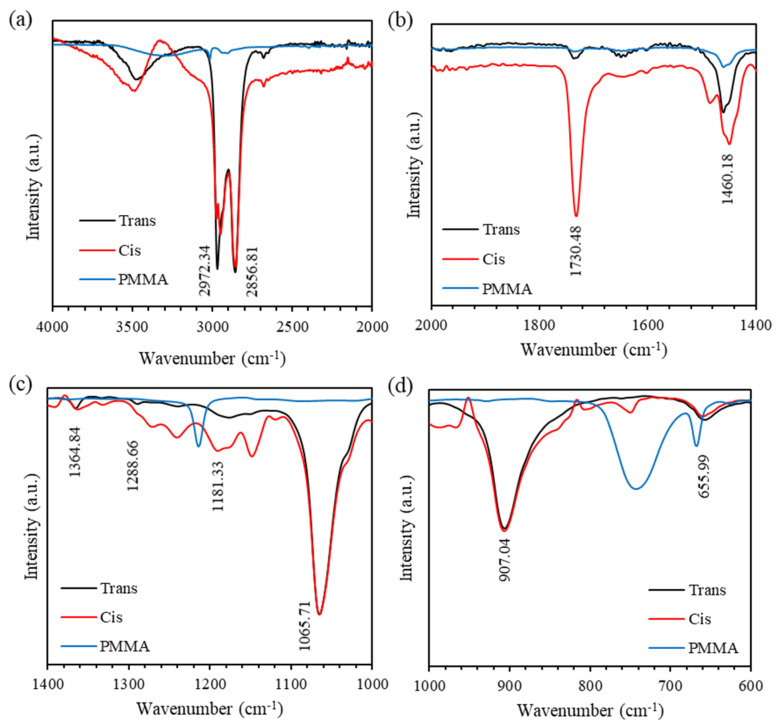
The FTIR spectra of *trans-* and *cis-*states of PMMA-BDK-MR polymer composite thin film: (**a**) 2000–4000 cm^−1^, (**b**) 1400–2000 cm^−1^, (**c**) 1000–1400 cm^−1^, and (**d**) 600–1000 cm^−1^.

Fourier transform infrared spectroscopy (FTIR) is conducted to study the vibrational bands of the nanocomposite. The chemical formula of PMMA and BDK are (C_5_O_2_H_8_)_n_ and (C_16_H_16_O_3_), respectively, while MR has a chemical formula of (C_15_H_15_N_3_O_3_). Figure 12 shows FTIR spectra of *trans-* and *cis-*states of PMMA-BDK-MR polymer composite thin film compared with PMMA thin film in the spectral regions: (a) 2000–4000 cm^−1^, (b) 1400–2000 cm^−1^, (c) 1000–1400 cm^−1^, and (d) 600–1000 cm^−1^. Figure 12a shows the peaks observed at 2972.37 cm^−1^ and 2856.81 cm^−1^ are assigned for -CH_2_- for PMMA and C-H bond stretching vibrations of the -CH_3_ group of PMMA, BDK, and MR, respectively. Figure 12b shows the peaks observed at 1730.48 cm^−1^ and 1460.18 cm^−1^ are dispensed for N = N for MR and asymmetric bending vibration C-CH_3_ stretching for PMMA, respectively. The peaks observed at 1181.33 cm^−1^ and 1288.66 cm^−1^ are allocated for C-O bond stretching vibrations of the -CH_3_ group for PMMA, BDK, and MR, while the peaks observed at 1364.84 cm^−1^ and 1065.71 cm^−1^ are associated with C-N in MR and bending O-CH_3_ in PMMA and BDK, respectively, as shown in Figure 12c. Finally, the peaks observed at 655.99 cm^−1^ and 907.04 cm^−1^ are apportioned for C-H bending and -C-O-C- in PMMA and BDK, as shown in Figure 12d. The IR spectra of thin film samples agree well with previous published findings and most relevant bands are listed with corresponding vibrational bands. Figure 12 shows that all band frequencies of both *cis*- and *trans*-MR are almost identical. The observation confirms that such interactions have only minor effects on at least the N = N frequencies at 1730.48 cm^−1^. The calculated IR intensity of N = N of *cis*-MR is extremely strong compared to the intensity of *trans-*MR, confirming our results in WRE optical data storage cycle.

### 4.4. Scanning Electron Microscope (SEM)

**Figure 13:**
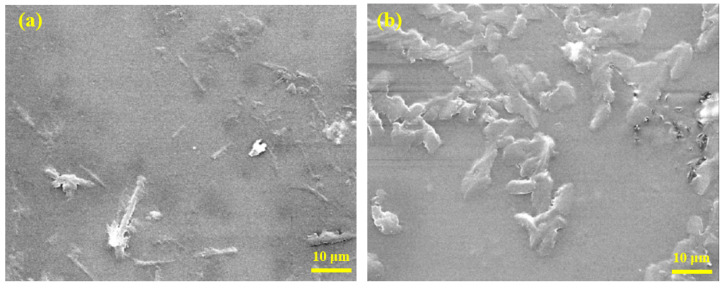
The SEM micrographs of PMMA-BDK-MR polymer composite thin films: (**a**) without curing and (**b**) with curing [48].

To elucidate the morphological features of the polymerized thin films, SEM measurements are performed. Figure 13 shows the SEM micrograph of PMMA-BDK-MR films before and after curing. In both cases, films exhibit amorphous nature. Before UV curing, the surfaces of films appear to be inhomogeneous compared with the surfaces of films after curing as can be clearly seen in Figure 13a,b. Exposing films to UV curing induces strong film polymerization, yielding surfaces that are highly homogenous. The random distribution of the inserted BDK into PMMA-host results in surfaces that are extremely inhomogeneous. However, illuminating films with UV light triggers BDK components of the composite to rearrange, occupying ordered sites throughout the PMMA matrix. The relaxation of different composite components to satisfy minimum energy requirement smoothen the surfaces greatly.

## 5. Conclusions

In summary, the underlying mechanisms of the kinematics of photoisomerization processes of PMMA-BDK-MR polymer composite thin films are investigated, analyzed, and interpreted. Elucidating the mechanisms is critical for optical application such as Read/Write/Erase (WRE) optical data storage media for the UV light Read/Write heads or UV light sensors. Deliberately, we add Azo Dye MR to the composite to give the solution the desired pH value and to accomplish the *cis ↔ trans* cycles through illumination *↔* thermal relaxation. The optical, chemical, and morphogical characterization of *trans*- and *cis*-states of PMMA-BDK-MR polymer composite thin films are performed using UV–Vis spectroscopy, Fourier transform infrared spectroscopy (FTIR) and scanning electron microscope (SEM). Furthermore, the obtained results are explored to investigate the possibility of using the PMMA-BDK-MR polymer composite thin films as an optical data storage media or optical and thermal sensors. The WRE cyclic process of conveying the electrochemical bonding form *trans* to *cis* and vice versa is investigated. We found that photo isomerization is initiated upon illuminating thin films with UV light of proper wavelength, transforming the films from *trans* to *cis* configuration. PMMA-BDK-MR polymerized thin films cured with 366 nm UV-light for 12 s is found to undergo *trans* ↔ *cis* cycles, successfully indicating the feasibility and validity of using photoisomerized thin films for optical data storage endeavors. In addition, a *cis*-form can be transformed back to a *trans*-form by means of thermal relaxation process. Remarkably, repeated *trans* ↔ *cis* cycles can be utilized for memory WRITE and memory READ applications. Finally, the morphology of the thin film surface is investigated using SEM technique. We found that surfaces of PMMA-BDK-MR thin films treated with UV light curing are homogenous in comparison with untreated surfaces. The transformation from inhomogeneous surfaces to homogeneous surfaces is attributed to the polymerization of thin films by UV curing. Having obtained *trans* ↔ *cis* cycles that can be repeated quickly is very important for proper functioning of photonic and optoelectronic devices based on polymerized thin films.
